# Correction: Choe et al. Effect of Ethics Seminar on Moral Sensitivity and Ethical Behavior of Clinical Nurses. *Int. J. Environ. Res. Public Health* 2021, *18*, 241

**DOI:** 10.3390/ijerph192013531

**Published:** 2022-10-19

**Authors:** Kwisoon Choe, Sunman Kim, Chunbok Lee, Sunghee Kim

**Affiliations:** 1 Department of Nursing, Chung-Ang University, 84 Heukseok-ro, Dongjak-gu, Seoul 06974, Korea; 2 Chung-Ang University Hospital, 102 Heukseok-ro, Dongjak-gu, Seoul 06973, Korea; 3 Institute for Historical Studies, Chung-Ang University, 84 Heukseok-ro, Dongjak-gu, Seoul 06974, Korea

The authors wish to make corrections to the order of the authors [1], and the affiliation information should also be re-ordered.

In the published paper, the order of the author’s names is: Chunbok Lee ^1^, Sunman Kim ^2^, Kwisoon Choe ^3^ and Sunghee Kim ^3,^*.

The correct order of the author’s names should be: Kwisoon Choe ^1^, Sunman Kim ^2^, Chunbok Lee ^3^ and Sunghee Kim ^1,^*.

In the published paper, the affiliations are in the order of:

1. Institute for Historical Studies, Chung-Ang University, 84 Heukseok-ro, Dongjak-gu, Seoul 06974, Korea;
2. Chung-Ang University Hospital, 102 Heukseok-ro Dongjak-gu, Seoul 06973, Korea;
3. Department of Nursing, Chung-Ang University, 84 Heukseok-ro, Dongjak-gu, Seoul 06974, Korea.

The correct order for the affiliations is:

1. Department of Nursing, Chung-Ang University, 84 Heukseok-ro, Dongjak-gu, Seoul 06974, Korea; kwisoonchoe@cau.ac.kr;
2. Chung-Ang University Hospital, 102 Heukseok-ro Dongjak-gu, Seoul 06973, Korea; ksm0517@caumc.or.kr;
3. Institute for Historical Studies, Chung-Ang University, 84 Heukseok-ro, Dongjak-gu, Seoul 06974, Korea; lcbpk@cau.ac.kr.

The authors apologize for any inconvenience caused and state that the scientific conclusions are unaffected. This correction was approved by the Academic Editor. The original publication has also been updated.
